# Investigating the role of utilitarian and hedonic goals in characterizing customer loyalty in E-marketplaces

**DOI:** 10.1016/j.heliyon.2023.e19193

**Published:** 2023-08-16

**Authors:** Ira Puspitasari, Febdian Rusydi, Nania Nuzulita, Chin-Sung Hsiao

**Affiliations:** a Information System Study Program, Faculty of Science and Technology, Universitas Airlangga, Surabaya, Indonesia; b Department of Physics, Faculty of Science and Technology, Universitas Airlangga, Surabaya, Indonesia; c Research Center for Quantum Engineering Design, Faculty of Science and Technology, Universitas Airlangga, Surabaya, Indonesia; d Department of Computer Science and Information Engineering, College of Information and Electrical Engineering, Asia University, Taichung City, Taiwan

**Keywords:** e-marketplace customer loyalty model, Hierarchical component model, Customer loyalty, Consumer behavior, Machine learning

## Abstract

Despite significant growth in sales in recent years, retaining customers remains a major challenge for the electronic marketplace (e-marketplace) industry worldwide, including Indonesia. The small basket size of Indonesian customers has created a highly price-sensitive market, making it difficult to nurture customer loyalty. This study investigates the factors affecting consumer behavior and loyalty in Indonesian e-marketplaces by employing a newly proposed E-Marketplace Customer Loyalty Model. Since customers' expectations have extended from mostly utilitarian-oriented goals to incorporate hedonic goals, the proposed model includes both utilitarian and hedonic-based constructs by integrating the Expectation-Confirmation Theory, e-service quality, and the Hedonic Information Systems Model. The proposed model was tested using a comparative approach of machine learning classification algorithms and partial least square, aiming to create a more robust model. The PLS analytical results of ECLM hiearchical component model from 678 customers show that the fulfillment of hedonic values via perceived enjoyment has a greater impact on customer satisfaction than utilitarian values of perceived service quality. Perceived enjoyment, personalization, and customer satisfaction positively affect customer loyalty. The classification results provide further evidence for all hypothesized relationships of the ECLM. The Sequential Minimal Optimization (SMO) algorithm has demonstrated superior performance compared with other classifiers in predicting the dependent variable in most cases. Based on findings, this study offers theoretical and practical implications, and recommendations for sustainable loyalty programs in e-marketplaces.

## 1 Introduction

The growth of online shopping transactions, including in e-marketplaces, has drastically increased since the outbreak of the COVID-19 pandemic. Initially, this surge occurred because of movement restrictions and lockdowns, which later induced changes in consumer-spending habits [[1], [2], [3]]. In line with global e-marketplace growth, Indonesia reported increasing use of e-marketplaces among users aged 16–64 years (85.4% of the total Internet users) [4]. This growth is due to the rising youth population, digital literacy, middle-class populations, digital payment services, and SMEs’ participation. Hence, among the biggest five e-marketplaces in Indonesia, two are Indonesian-based, i.e., Tokopedia and Bukalapak. In addition, the COVID-19 pandemic in Indonesia has contributed to the increased transactions on major e-marketplace platforms. Many users shifted to online platforms since most provinces and cities imposed large-scale social restrictions, and they continued using the e-marketplace after the pandemic slowdown.

With the growth of online shopping and e-marketplaces, the market has become increasingly competitive. This competition is due to the relatively small basket size of Indonesian customers at $36 (compared to its neighboring countries, Malaysia at $54, and Singapore at $91) [5], hence, most customers are price-sensitive and tend to seek cheaper deals. Companies that use promotional pricing strategies face the danger of depreciation, such as devaluing their products. Customers may believe that a product is too expensive, which will have a negative effect on profit and product/service quality. In addition, this strategy may fail to gain customer loyalty, which is essential for business survival and success, because attracting new customers is more challenging and expensive than retaining existing ones. Thus, maintaining loyalty is challenging in a competitive market like the e-marketplace industry.

Since it is more expensive and challenging to attract new customers than to retain existing ones, loyalty is crucial to business survival and success. Hence, maintaining loyalty is challenging in a competitive market, such as in the e-marketplace industry, among price-conscious customers, and is an unprecedented situation like the COVID-19 pandemic. Any businesses, including e-marketplace providers, are recommended to go above and beyond studying their customers’ behavior and loyalty factors to grow better. Hsu (2008) proposed an online customer satisfaction index (e-CSI) to predict customer loyalty and satisfaction among Taiwanese customers [6]. Other studies have demonstrated the positive impact of service quality on customer loyalty [[7], [8], [9], [10]]. Furthermore, corporate image, satisfaction, complaint handling, and trust play important roles in predicting customer loyalty in the business-to-business (B2B) sector [11].

Customer satisfaction plays a vital role in customer loyalty. When customers are satisfied with their experiences, the chances of them repurchasing and continuing the business relationships are high. In a study of online banking continuance intention based on a combination of Expectation Confirmation Theory (ECT) and self-determination theory, intrinsic regulation and customer satisfaction are the most important factors in determining continuance intention [12]. Another study on the application of ECT in e-wallet usage suggested that confirmation has a positive impact on satisfaction and continuance intention [13]. In mobile e-commerce platforms, perceived usefulness and playfulness (perceived performance), and confirmation positively impacted customer satisfaction; and customer satisfaction and perceived playfulness significantly affect the continuance intention to use e-commerce [14].

As many customers now feel safe shopping online, their goals have extended from mostly utilitarian-oriented to more hedonic. A study on mobile Internet adoption suggests that the fulfillment of hedonic aspects through perceived enjoyment positively influences users’ continuance intention to use the technology [15]. Concurrently, other studies reported that perceived enjoyment positively determines repurchase intention [16,17], post-adoption satisfaction and loyalty [18], as well as mobile apps adoption and m-loyalty [19].

This study aims to investigate the factors affecting customer behavior and loyalty in using e-marketplaces. Customers in this study are buyers who purchase products and services from official stores and/or sellers in the e-marketplace. Therefore, understanding customer behavior and the factors driving their loyalty allows e-marketplace providers to develop more useful and effective strategies for customer retention. From a theoretical perspective, this study extends the current literature on consumer studies, particularly in the e-marketplace context, by characterizing their perceived service quality (PSQ); uncovering the linkages between utilitarian-based constructs, hedonic-based constructs, as well as the customers’ post-purchase evaluation.

## 2 Literature background

Previous studies have investigated the factors affecting customer satisfaction and loyalty in various e-commerce systems, such as online stores [20,21], specific e-commerce platforms [[22], [23], [24]], and online shopping systems [25,26]. The shift to online services was predominantly driven by utilitarian-oriented goals to fulfill customers' specific needs and achieve specific outcomes efficiently and effectively [[6], [7], [8],^10^,^21^,^25^,^27^]. The most common approach to examine how customers form and assess the fulfillment of their utilitarian goals based on their pre-existing expectations and post-consumption experiences is Expectation Confirmation Theory (ECT) [28,29]. This theory emphasizes the role of expectations in shaping customer satisfaction judgments and continuance use. Hence, customers' subjective assessment of system quality determines their post-consumption behavior. When the performance of an online service system surpasses the customer's initial expectations, it leads to positive confirmation that subsequently enhances post-adoption satisfaction [28,30]. This subjective assessment of perceived usefulness is closely tied to the customer utilitarian goal.

Previous studies have adopted the concept of perceived usefulness in ECT to examine customer and user post-adoption behaviors in e-commerce systems [6,11,20,21,25]. Because the industry is highly competitive, these companies must provide excellent services to attract customer attention, which leads to long-term relationships. The adoption of perceived usefulness in these systems has expanded to include more comprehensive service quality attributes [6,11,24,31]. E-service quality refers to the degree to which e-commerce facilitates efficient and effective online transactions through shopping, purchasing, and delivery [24,26,[31], [32], [33]]. In a study of the online customer satisfaction index (e-CSI), Hsu et al. (2008) proposed five e-SQ attributes: information availability and content, ease of use, privacy/security, graphic style, and fulfillment/reliability [6]. This study also found that the proposed e-SQ positively influenced loyalty through the mediation of customer satisfaction. Blut (2016) proposed a third-order e-service quality model with four dimensions: website design, fulfillment, customer service, and security/privacy [33]. In another study, Sheu and Chang (2021) defined service quality in four dimensions–fulfillment, efficiency, system availability, and privacy–to study the relationship between service quality, satisfaction, and loyalty in Shopee e-commerce [24]. The results showed that all service quality dimensions were positively associated with customer satisfaction, whereas fulfillment, efficiency, and privacy significantly affected loyalty. In a study on the role of e-service quality on customer loyalty online, the proposed e-service quality had two dimensions: information security and website performance shopping [34]. The study found that all dimensions were positively associated with e-service quality, and e-service quality was positively associated with e-loyalty.

Expectation-Confirmation Theory (ECT) states that confirmation of expectations evaluates the consistency between user expectations of Information Technology (IT) systems and actual usage, which implies the realization of the expected benefit from the use of IT systems. Positive confirmation occurs when actual experiences align with or surpass users' initial expectations [35,36]. Subsequently, the inline or exceeded realization of perceived service quality from the actual use forms and enhances user satisfaction [10,24,26,35,36]. In addition, a positive confirmation also leads to satisfaction as it validates users’ initial expectations, creates a sense of fulfillment, and generates positve emotions. Previous studies in e-commerce systems have confirmed the positive influence of confirmation of expectations on user satisfaction [6,11,27,36].

As the use of e-commerce systems, including e-marketplaces, has become habitual for many users, their expectations have expanded beyond utilitarian-oriented goals. Users expect shopping activities to fulfill their utilitarian goals and deliver hedonic experiences [16,19,20,37,38]. One of the core manifestations of hedonic experiences in shopping activities is the users’ perception of enjoyment. Past studies have demonstrated the positive association between the confirmation of user expectations and perceived enjoyment [35,39,40]. The attainment of perceived enjoyment in shopping activities generates positive emotional associations, increases engagement, and enhances customer satisfaction [21,38,41,42], contributing to loyalty in long-term relationships [16,19,37].

In a highly competitive e-commerce landscape, customers are on the upper hand because they have various choices. Satisfaction with the initial purchase determines post-purchase events such as behavior change, engagement, and loyalty. As satisfaction increases, customers are more likely to be loyal and repurchase or recommend and unconsciously advise the products/services to others [6,10,11,24,36,[43], [44], [45]]. In the e-commerce industry, satisfaction alone does not guarantee repeated transactions and continued use of e-commerce. The digital nature of e-commerce systems allows customers to compare prices, product offerings, and experiences across different platforms at their fingertips. This transparency empowers customers to make informed decisions and swich to platforms that offer better deals or superior experiences. In addition, the numerous available e-commerce platforms and the minimal switching cost raise customers expectations for even higher service quality. One common strategy to retain customers is to offer personalized services in many aspects of their interactions with systems. Personalization increases the delivery quality of utilitarian and hedonic goals, fosters brand attachment, and ultimately helps build long-term relationships [38,44,46,47].

Table 1 describes the operational definition of constructs based on a literature study of the factors affecting customer loyalty in e-marketplaces.

**Table 1:** Operational definition of construct.

Construct	Definition	Adapted From
Perceived service quality (PSQ)	The extent to which an e-marketplace facilitates the fulfillment of utilitarian-oriented goals that is associated with all customers' interactions with the e-marketplace, including product and information search, the purchasing and delivery process, customer service interactions, and security and privacy handling. The perceived service quality of e-marketplace in this study is a hierarchical construct formed by its five lower order dimensions: system design, fulfillment, customer service, security, and efficiency.	[26,[31], [32], [33],^43^]
Confirmation	Consistency between customers' expectations with actual experiences toward the use of e-marketplaces.	[6,28,35,36]
Perceived Enjoyment	Customers' perceptions of the enjoyable and pleasurable aspects of using an e-marketplace, that align with their hedonic motivations.	[21,25,35,38,48]
Satisfaction	The customer's level of overall satisfaction after evaluating the actual service quality delivered by e-marketplaces against the expected performance.	[6,11,26,28]
Personalization	The degree to which the e-marketplace services and offerings is well tailored to meet specific individual needs and preferences of each customer.	[38,44,46,47]
Customer Loyalty	The degree of attachment, commitment, and repeat purchase behavior exhibited by customers towards a specific e-commerce platform.	[6,11,28,36]

## 3 Conceptual framework and hypotheses development

This study proposes the E-marketplace Customer Loyalty Model (ECLM) adapted from the Expectation-Confirmation Theory [28,30], e-service quality [33,49], as well as User Acceptance of Hedonic Information Systems [50] to examine the factors contributing to customer loyalty in e-marketplaces. ECLM includes utilitarian- and hedonic-based constructs, since both are determinants of the adoption and continuance use of e-marketplaces [20,21,25,42]. The utilitarian aspect focuses on the achievement of predetermined goals resulting from cognitive behavior [51]. For instance, a customer uses an e-marketplace to seek and evaluate items before purchasing, compares prices, and purchases items instantly. Hedonic motivation underlies the reactive behavior in handling the emotional aspect of the shopping experience [21].

Covering the utilitarian aspect in the ECLM model, ECT posits that user satisfaction is directly influenced by confirmation of expectations from system usage and perceived usefulness. When the performance of a system exceeds the user's initial expectation, the positive disconfirmation increases post-adoption satisfaction. Next, satisfaction is a function of multiple attributes related to system and service quality. In this study, we propose perceived service quality (PSQ) as a utilitarian antecedent of customer satisfaction in the e-marketplace. The proposed PSQ construct assesses customers' expectations of self-service systems using four dimensions adapted from e-service quality [33] and the efficiency dimension. This study considers the PSQ a hierarchical construct formed by five lower-order dimensions. The hierarchical conceptualization of general service quality is deemed more suitable for accommodating the complexity of human perceptions [33,43,52]. Customers initially evaluate the service quality of e-marketplace systems from a specific element at the individual dimension level and then synthesize their judgments to a more general construct. The PSQ's dimensions in this study are as follows.

(1) System design encompasses all aspects of customer interaction with the e-marketplace system, such as the system's usability, purchase process, navigation, and availability [31,53]. Since the e-marketplace system is becoming more complex, customers demand a straightforward purchase process (for example, a buy-now feature, and responsive design implementation) to expedite frequent purchases. Ease of use is also a crucial factor because customers shift to online shopping for convenience, and they expect problem-free shopping experiences.
(2) Fulfillment refers to all back-end processes from receiving, processing, and delivering customer order [33,54]. The fulfillment quality includes the delivery time, order accuracy, billing accuracy, and truthfulness of the offerings. Poor order fulfillment can jeopardize customer retention.
(3) Security includes safeguarding payment systems, preventing fraud and financial loss, and protecting personal data [26,33]. As personal and financial data must be provided while shopping online, security is a crucial factor to consider when a customer is using e-marketplaces.
(4) Customer service includes the service level and assistance in making purchase decisions, resolving issues, and managing returns. In an e-marketplace, customer service handles inquiries before, during, and after sales. The responsiveness of customer service contributes highly to the overall PSQ [31]. Accordingly, e-marketplace industries provide synchronous media across channels and platforms for customer services, such as live chats, online helpdesks, and social networks.
(5) Efficiency involves the accessibility aspect, which corresponds to how the system presents its content and responds to customers' actions, and the availability aspect, which refers to the required technical functions and the accuracy of the service. Notably, efficiency has been validated in previous studies as one of the required constructs to assess online business' service quality like in mobile service quality [54], as well as e-service quality in B2B e-marketplaces [31].

Previous studies have reported that the utilitarian attribute positively influence user satisfaction like the performance expectancy in mobile applications [27], e-service quality in online shopping [24,26,36], and service quality in a retail banking service [43]. Similarly, positive confirmation occurs when customers believe that the e-marketplace provides excellent service quality [35,36]. Therefore, a greater degree of customers’ PSQ enhances the levels of their confirmation of e-marketplace usage. Concurrently, previous research supports the following hypotheses:

H1 PSQ is positively associated with customer satisfaction.

H2 PSQ is positively associated with customers' extent of confirmation of e-marketplaces use.

The confirmation of expectations has been considered to be one of the key cognitive factors of user satisfaction and continuance use of IT systems. Customers’ confirmation of expectations implies that they receive the expected benefits of using the e-marketplace, increasing their level of satisfaction. Previous research based on ECT has confirmed the positive influence of confirmation on overall satisfaction [6,27,29,36]. However, since the use of an e-marketplace also incorporates hedonic-oriented goals, the actual user experience will confirm the previous expectations of perceived enjoyment. Previous studies have shown the positive influence of confirmation of expectations on perceived enjoyment [18,19,35,39]. Thus, we propose the following hypotheses:

H3 Customers' extent of confirmation is positively associated with their satisfaction with e-marketplaces use.

H4 Customers' extent of confirmation is positively associated with their perceived enjoyment in using e-marketplaces.

Recent studies have highlighted the significant influence of perceived enjoyment on the acceptance of an IT system for both utilitarian/organizational and hedonic purposes [18,40,48,55,56]. This construct was derived from the User Acceptance of the Hedonic Information Systems model. Perceived enjoyment emphasizes the fun aspects of adopting IT systems. Based on TAM (total available market) meta-analysis, perceived enjoyment is a key factor in the adoption of innovative systems and entertainment services [55]. In the e-marketplace industry, developers and executives often release and update entertainment features (gamified campaigns, mini-games for promotion, interactive content, and other add-on offers) to attract and retain customers. Hence, shopping in the e-marketplace provides instrumental values and pleasurable experiences. Perceived enjoyment is expected to significantly affect the development of post-adoption satisfaction and loyalty, as confirmed in previous studies [16,19,21,37,38,41,42]. Thus, we hypothesize:

H5 Customers' perceived enjoyment is positively associated with their satisfaction in using e-marketplaces.

H6 Customers' perceived enjoyment is positively associated with their loyalty to e-marketplaces.

Customer loyalty is primarily determined by satisfaction with prior usage/experiences. In the context of e-marketplace, customer loyalty refers to the continuance intention to use e-marketplace for utilitarian and hedonic goals and to recommend the e-marketplace to others. To this point, previous studies have confirmed that satisfaction is the key influence on loyalty and future usage intention [6,10,16,24,36,[43], [44], [45]]. Accordingly, we propose the following hypothesis:

H7 Customers satisfaction is positively associated with their loyalty to e-marketplaces.

Once customers have become accustomed to the shopping process in an e-marketplace, they tend to do all purchases in a particular e-marketplace because it is more convenient and time-efficient for them. Additionally, e-marketplaces offer an assortment of loyalty programs that benefit the customers. A proven strategy to increase customer satisfaction is to accommodate personalization in the e-marketplace system based on customers' preferences and usage history. Previous studies have also highlighted the positive influence of personalization on customer loyalty [38,44,46,47]. Thus, this proven strategy leads to the following hypothesis:

H8 System personalization is positively associated with customer loyalty to e-marketplaces.

**Fig. 1:**
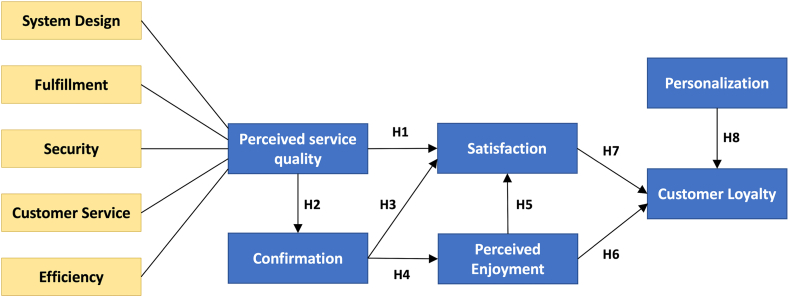
The research model*:* E-marketplace Customer Loyalty Model.

Fig. 1 shows the ECLM based on the proposed hypothesis.

## 4 Materials and method

### 4.1 Questionnaire design

The data collection instruments consisted of a demographics profile and an e-marketplace usage section, as well as a questionnaire containing 32 questions to examine customer loyalty to the e-marketplace. The questionnaire was adopted from the previously validated studies on ECT, the e-service quality model, and the User Acceptance of the Hedonic Information Systems model. Responses in the questionnaire were six points Likert scales ranging from strongly disagree (scale 1) to strongly agree (scale 6). This study used a six-point (even) Likert scale, without including a neutral option, to encourage participants to be more selective and thoughtful with their responses. The absence of a neutral option eliminates the tendency to gravitate towards a neutral response, as the topic of the e-marketplace is not controversial. Another problem with the neutral option is its misuse for reasons other than expressing neutrality. This misuse could potentially impact the validity of the study results [[57], [58], [59], [60]].

The questionnaire was written in English and Bahasa Indonesia, and professional translators translated the questionnaire from English to Bahasa Indonesia and all responses from Bahasa Indonesia to English. The questionnaire items and the corresponding measurement of latent constructs are presented in Table 2.

**Table 2:** Measurement of latent constructs and questionnaire item.

Construct	Indicator	Questionnaire Item	Adapted From
System Design (SYD)	SYD1	The use of the e-marketplace is clear and understandable.	[31,33,61]
	SYD2	I can complete my purchase order easily, from searching a product, making a payment, to confirming transaction.	
	SYD3	It is easy to navigate the interface of e-marketplace website/application and find items/features that I am looking for.	
Fulfillment (FUL)	FUL1	The e-marketplace ships orders within a reasonable timeframe.	[33,54,61]
	FUL2	The logistics system of this e-marketplace provides enough options for me.	
	FUL3	The e-marketplace provides a guarantee for order fulfilment.	
	FUL4	The e-marketplace is truthful about its offers (e.g., price discount, cashback, and shipping fee reduction).	
Security (SEC)	SEC1	The e-marketplace protects my financial information.	[33,54]
	SEC2	The e-marketplace protects my personal information, such as personal details and purchasing behavior.	
	SEC3	The e-marketplace system has adequate security features.	
Customer Service (CUS)	CUS1	The e-marketplace provides a dedicated customer support to attend to any questions or comments that I might have.	[31,61]
	CUS2	The e-marketplace resolves my problems promptly.	
	CUS3	The e-marketplace provides convenient options for returning items.	
Efficiency (EFF)	EFF1	The e-marketplace system loads its content quickly.	[31,54,61]
	EFF2	The e-marketplace response to my actions quickly.	
	EFF3	The e-marketplace system is always available and ready to perform transactions whenever I access the system.	
Confirmation (CON)	CON1	My experience with using the e-marketplace is better than what I expected.	[6,18,28]
	CON2	The service level provided by the e-marketplace is better than what I expected.	
	CON3	The majority of my expectations from using e-marketplace are confirmed.	
Perceived Enjoyment (PEN)	PEN1	I enjoy exploring the e-marketplace features (e.g., exploring the flash sale, participating in a mission for rewards, using vouchers and coupons for additional benefits).	[18,40,62]
	PEN2	I enjoy shopping in the e-marketplace.	
	PEN3	Overall, my experiences of using the e-marketplace are enjoyable.	
Customer Satisfaction (SAT)	SAT1	The e-marketplace always meets my needs.	[18,27]
	SAT2	Overall, I am satisfied with this e-marketplace.	
Personalization (PER)	PER1	The e-marketplace suggests items that suit my preferences.	[62]
	PER2	The promotion and advertisements in the e-marketplace are relevant to my shopping needs.	
	PER3	The personalization provided by the e-marketplace benefits me.	
Customer Loyalty (LOY)	LOY1	I recommend this e-marketplace to others (referrals).	[28,31,61]
	LOY2	I am likely to continue to use the marketplace in the future (repeat purchase).	
	LOY3	I intend to continue using the e-marketplace rather than discontinue its use.	

The preliminary questionnaire testing included content validity, a pilot test, and an internal consistency test. We invited four domain experts (two information system experts, one business expert, and one consumer behavior expert) and one psychometrician from various universities, and one employee from an e-marketplace to assess the relevancy and representativeness of each questionnaire's item to the consumer loyalty domain. Following the content validity assessment in this study, a systematic process was used [63]. All experts reviewed each item, provided written feedback, and scored the relevance of each item in the questionnaire using a degree of relevance scale. The scale consisted of four ratings: 1) the item is irrelevant, 2) the item is somewhat relevant and requires revision, 3) the item is relevant with minor revision, and 4) the item is highly relevant. Before calculating the Content Validity Index (CVI), ratings 1 and 2 were recategorized to 0, and ratings 3 and 4 were recategorized to 1. The CVI score was calculated on basis of formulations by Polit et al. [64]. The questionnaire's S-CVI/Average Variance Extracted (AVE) score of 0.96 was higher than the threshold CVI value of 0.83 [64], hence the questionnaire contents met the satisfactory level to represent the measured outcome of customer behavior and loyalty in the e-marketplace. However, some minor revisions were added to the original questionnaire according to the experts' comments.

A pilot test was conducted on a sample of 40 participants. Each participant was asked to complete the questionnaire; rated the relevancy of each item and the readability of the questionnaire; and shared their overall impression of the questionnaire. The final test, known as internal consistency, examined the correlation between items loaded onto the same factor and the consistency of participants' responses. Based on the pilot test results, Cronbach's alpha was 0.78, indicating acceptable internal consistency. After minor modifications following the participants' feedback, we distributed the final questionnaire for data collection.

### 4.2 Data collection and analysis

The participants in this study were Indonesian citizens and residents, aged 18 years or older, who had purchased items from at least one Indonesian-based e-marketplace on the web or mobile applications. Participants were recruited by email, social media invitation, and messaging apps, such as Telegram and WhatsApp, from May to June 2022 and August to September 2022. The questionnaire was administered online using a web-based survey system. Before beginning data collection, participants were asked to review a consent form. If the participants agreed to participate, the process proceeded to the filter question to assess their suitability. In case of disagreement, the data collection process was terminated. The filter question asked whether the participant had made at least one purchase transaction in any Indonesian e-marketplace. If the response was no, data collection was stopped, as the participant did not meet the criteria. Conversely, if the response was yes, the data collection continued to the demographics profile and e-marketplace usage sections. Finally, in the questionnaire section, participants were asked to answer 32 questions about their perceptions of customers using e-marketplaces.

The responses from all participants were compiled using descriptive statistical analysis, while the proposed ECLM model was tested using partial least square structural equation modeling (PLS-SEM). Given that one of the study goals was to identify the driver's construct, PLS-SEM was suitable for this confirmatory-explanatory study because the proposed model had many indicators, and the data were non-normally distributed [65,66]. Based on the participants' responses, we conducted the Shapiro-Wilk test to check the normality of our dataset [67]. The distributions of all constructs and PSQ dimensions were found to be significantly non-normal, including: system design (W = 0.7637, *p* < 0.001), fulfillment (W = 0.8778, *p* < 0.001), security (W = 0.8762, *p* < 0.001), customer service (W = 0.8731, *p* < 0.001), efficiency (W = 0.8676, *p* < 0.001), PSQ (W = 0.8761, *p* < 0.001), confirmation (W = 0.8356, *p* < 0.001), perceived enjoyment (W = 0.8056, *p* < 0.001), satisfaction (W = 0.8311, *p* < 0.001), personalization (W = 0.8446, *p* < 0.001), and customer loyalty (W = 0.8014, *p* < 0.001).

**Fig. 2:**
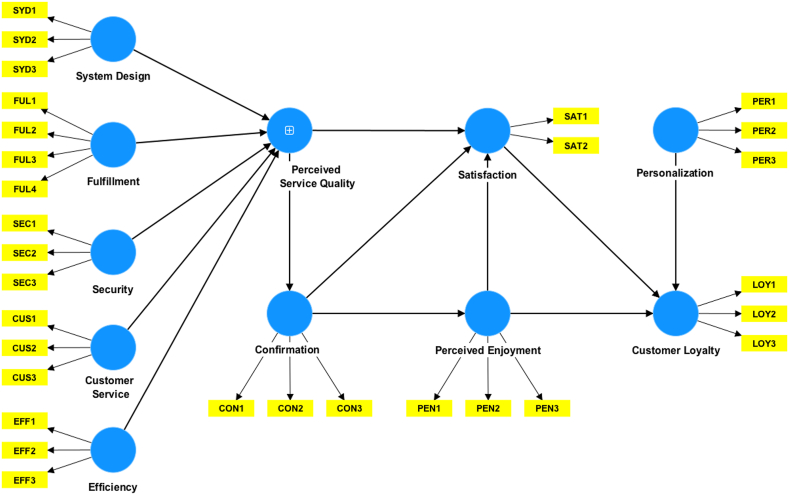
The PLS path diagram of hierarchical component model of ECLM. Perceived Service Quality (PSQ) is the second order construct.

The Extended Component-Level Model (ECLM) in this study employed a hierarchical component model type II, which incorporated formative relationships between lower-order components (LOCs) and higher-order components (HOCs), and reflective relationships between all first-order constructs and their indicators, as shown in the path diagram in Fig. 2. PLS-SEM model testing in this study followed the extended repeated indicators approach [68,69]. The first stage analyzed the measurement models of the reflective LOCs. The measurement model assessed the indicator and internal consistency reliability, convergent, and discriminant validity of the proposed constructs [69]. First, the indicator reliability is examined by its outer loadings value. The recommended threshold for outer loadings value should be higher than 0.7 [69]. Internal consistency evaluates the reliability based on the correlation of the observed constructs. PLS uses Composite Reliability (CR) to measure internal consistency and its recommended value ought to be higher than 0.7 [69]. Convergent validity refers to the degree to which the measures of the expected related constructs are highly correlated. The convergent validity is satisfied when each item has outer loadings above 0.70 and each construct's AVE index is 0.50 or higher [69]. Next, discriminant validity refers to the extent to which a construct is empirically different from the other constructs in the model [69]. One approach to check discriminant validity is to use the heterotrait-monotrait ratio of correlations (HTMT) [70]. A study by Henseler et al. showed that the specificity and the sensitivity rates of HTMT measurement were higher than those of the cross-loadings criterion and Fornell-Lacker method [70]. The threshold value of HTMT is 0.85, as suggested by Kline RB [71], and 0.9 as proposed by Gold et al. [72], and a value above this threshold implies a lack of discriminant validity. The last step was calculating the latent variable (LV) scores for all LOCs.

The second stage continued with the measurement model of HOCs. Following the extended repeated indicators approach [68,69], the latent variable scores of the PSQ dimensions were used as manifest variables for the PSQ construct in the HOC measurement model. The PSQ was evaluated at both dimension and construct levels. At the dimension level, the multicollinearity test, the significance and sign test of outer weights were computed [43,68,69]. The multicollinearity evaluation included the Variance Inflation Factor (VIF) and cross-loadings of indicators. VIF indicates the degree of collinearity between the latent constructs. In the context of PLS-SEM, a VIF value exceeding 5 suggests the presence of potential collinearity issues [69]. If the VIF value is below the threshold, the analysis proceeds to assess the outer weights' significance and relevance.

Next, the structural model evaluation examined the overall fit of the estimated model, coefficient of determination (*R*^*2*^), effect sizes (f^2^), and path coefficient estimates of HOCs. In the extended repeated indicator approach, LOCs are not considered part of the structural model [68]. The *R*^*2*^ explains the share of variance of an endogenous construct, hence it measures the model's predictive accuracy. The effect size *f*^*2*^ measures the relationships between constructs, with *f*^*2*^ values below 0.02, 0.02–0.150, 0.150–0.350, and larger than 0.350, exhibiting no substantial, weak, medium, and large effect sizes [73]. The relationships among hypothesized latent constructs and observable variables are examined based on the path coefficient estimates and their significance levels evaluation.

In an attempt to develop a more robust model, we adopted a comparative analytical approach by integrating machine learning classification into data analysis [74]. Predictive models based on machine learning have been extensively utilized in consumer research and social sciences to characterize and forecast consumer behavior [75]. The predictive classification models in this study were employed to either support or contradict the results of hypothesis testing. We implemented five classifiers to characterize and predict consumer behavior based on the proposed ECLM. These classifiers included a Bayesian classifier (Naïve Bayes), an optimization algorithm that iteratively solves the Support Vector Machine's quadratic programming (Sequential Modeling Optimization), an implementation of the k-nearest neighbors algorithm (Instance-based learning, IBk), a rule-learner (OneR), and a decision tree (Random Forest). The performance of the classifiers was assessed based on several metrics, including correctly classified instances (accuracy), precision, recall, F1 score, and receiving operator characteristics (ROC) area.

## 5 Results

This section reports the participants’ descriptive profiles, measurement model assessment, structural model evaluation, and hypothesis testing results using machine learning classifiers.

### 5.1 The profile of the participants

A total of 1126 participants from Indonesia contributed to the data collection, following the dissemination link through email, messaging apps, and social media; however, only 713 participants completed the questionnaire completely (63.32% response rate). Of these participants, 35 responses were discarded because they were invalid and inconsistent, resulting in a final sample of 678 valid responses. This gender profile was in line with prior studies on online shopping behaviors, which reported that more than half of the participants were female [76]. One of the reasons why women made the majority of consumer purchases is because of the role of being a caregiver in the family and society. We also performed a permutation test [69] to check for response bias between the gender groups. The results showed that the difference between the responses of males and females was not significant. It suggested that the evidence did not support the presence of a response bias due to the inequality of responses between the two groups. More than half of the participants were aged between 18 and 25 years, followed by the 26–35 age group. Most younger adults are digital natives and they have accustomed to shopping online. However, despite the increasing number of Internet users in Indonesia, connectivity is still highly concentrated on Java. The geographical distribution shows that more than 60% of the participants lived on Java. Table 3 presents the demographic characteristics of the participants.

**Table 3:** Demographics characteristics of the participants.

Category	n (%), N = 678
Gender	
Male	273 (40.27)
Female	405 (59.73)
**Age Group (in years)**	
18–25	348 (51.33)
26–35	195 (28.76)
36–45	117 (17.25)
46–55	13 (1.92)
>55	5 (0.74)
**Occupation**	
Student: high school/undergraduate/postgraduate	169 (24.93)
Civil servant	83 (12.24)
Private employee	229 (33.76)
Professional	47 (6.93)
Entrepreneur	60 (8.85)
Homemaker	53 (7.82)
Others	37 (5.46)
**Geographical Distribution**	
The Greater Jakarta, area including Jakarta, Bogor, Depok, Tangerang, and Bekasi cities	153 (22.57)
Java (outside The Greater Jakarta) and Madura	284 (41.89)
Bali and Nusa Tenggara	48 (7.08)
Sumatra	96 (14.16)
Kalimantan	41 (6.05)
Sulawesi	39 (5.75)
Maluku and Papua	6 (0.88)
Others	11 (1.62)

Most participants have used e-marketplace for one to three years (see Table 4). The recent switch to online shopping must have been majorly influenced by the COVID-19 pandemic, where mobility restrictions were imposed to curb the spread of the disease. Additionally, customers expanded their purchases to include items that were not usually purchased online, such as fresh food, medicines, and groceries. Most participants reported using e-marketplaces one to five times per month, depending on their needs. Aside from purchasing items, more than half of the participants reported that they used e-marketplaces to top-up credits (e.g., prepaid phone credit and digital money) or pay bills (e.g., utilities, taxes, and insurance). Only a few participants purchased tickets for events or travel. The dominance of travel apps and the late addition of travel and entertainment ticket features to the e-marketplace could be a reason why these features are not popular. Most participants used more than one e-marketplace, with Shopee and Tokopedia being the most used (49.85% and 43.51%, respectively), while less than 7% of participants reported that their most frequently used e-marketplace was other than Shopee and Tokopedia. However, according to a recent report, Shopee and Tokopedia have the highest number of monthly visitors and downloads on Google PlayStore and AppStore [77]. Finally, the majority of participants selected promotional campaigns as the most attractive feature of their favorite e-marketplace (see Table 4). This demonstrates how significantly customers’ purchasing decisions are influenced by price sensitivity. Other significant factors were the ease of use and the large selection and variety of products.

**Table 4:** E-marketplace platform and usage.

Category	n (%), N = 678
E-marketplace platform(s) where participants had made at least one purchase*	
Tokopedia	522 (76.99)
Shopee	556 (82.00)
Bukalapak	99 (14.60)
Lazada	298 (43.95)
Blibli	60 (8.85)
Others	75 (11.62)
Most frequently used e-marketplace platform	
Tokopedia	295 (43.51)
Shopee	338 (49.85)
Bukalapak	7 (1.03)
Lazada	27 (3.98)
Blibli	2 (0.30)
Others	9 (1.33)
E-marketplace period of use	
Less than a year	42 (6.19)
1–3 years	431 (63.57)
4–6 years	189 (27.88)
More than 6 years	16 (2.36)
E-marketplace frequency of use	
As the need arises	334 (49.27)
1 - 5 times/month	224 (33.04)
6 - 10 times/month	50 (7.37)
11 - 15 times/month	43 (6.34)
>15 times/month	27 (3.98)
Shopping activities in e-marketplace *	
Purchase items	674 (99.41)
Top-up prepaid credits and pay bills	431 (63.57)
Purchase tickets for travel and entertainment	45 (6.64)
Access gamification features for additional benefits (e.g., discount, coupon, and cashback)	134 (19.76)
Watch entertainment content for additional benefits (e.g., discount, coupon, and cashback)	32 (4.72)
Reasons for shopping at e-marketplace *	
Large selection of available items	461 (67.99)
Promotional campaigns, e.g., discount, cashback, shipping fee reduction	570 (84.07)
Ease of use	468 (69.03)
Completeness of features	206 (30.38)
Aesthetics design and clear navigation	160 (23.60)
Brand ambassadors	46 (6.78)
Others	20 (2.95)

Note: * participants can select multiple answers.

### 5.2 Measurement model: first-order constructs with reflective indicators

SmartPLS4 software was used [78] to assess the reliability and validity of the proposed ECLM. As indicated in Table 5, the outer loadings values of all indicators were greater than the recommended threshold of 0.7, the composite reliability of each indicator exceeded 0.7, and the AVE of all constructs ranged between 0.646 and 0.838, exceeding the recommended value of 0.5. Therefore, indicator reliability, internal consistency, and convergent validity of the first-order constructs were validated.

**Table 5:** Outer loadings, internal consistency, convergent validity.

Construct	Indicator	Outer Loadings (>0.7)	Cronbach's α	CR^a^ (>0.7)	AVE^b^ (>0.5)
System Design (SYD)	SYD1	0.884	0.778	0.872	0.694
	SYD2	0.793			
	SYD3	0.819			
Fulfillment (FUL)	FUL1	0.789	0.816	0.879	0.646
	FUL2	0.812			
	FUL3	0.863			
	FUL4	0.746			
Security (SEC)	SEC1	0.898	0.836	0.901	0.753
	SEC2	0.803			
	SEC3	0.898			
Customer Service (CUS)	CUS1	0.858	0.795	0.880	0.709
	CUS2	0.849			
	CUS3	0.819			
Efficiency (EFF)	EFF1	0.868	0.818	0.891	0.733
	EFF2	0.865			
	EFF3	0.835			
Confirmation (CON)	CON1	0.883	0.856	0.913	0.777
	CON2	0.906			
	CON3	0.855			
Perceived Enjoyment (PEN)	PEN1	0.750	0.768	0.865	0.683
	PEN2	0.880			
	PEN3	0.844			
Satisfaction (SAT)	SAT1	0.908	0.806	0.912	0.838
	SAT2	0.922			
Personalization (PER)	PER1	0.824	0.841	0.904	0.760
	PER2	0.902			
	PER3	0.886			
Customer Loyalty (LOY)	LOY1	0.798	0.818	0.887	0.724
	LOY2	0.899			
	LOY3	0.851			

Note.

a Composite Reliability.

b Average Variance Extracted.

This study used the heterotrait-monotrait (HTMT) ratio of the correlations criterion to examine the discriminant validity. Table 6 shows that all constructs’ HTMT values were below 0.85. Thus, according to Refs. [70,72], the discriminant validity between the two constructs in this study was confirmed.

**Table 6:** Discriminant validity test: Heterotrait-monotrait (HTMT) ratio of correlations criterion.

	CON	LOY	SAT	CUS	FUL	PEN	PER	EFF	SEC	SYD
CON										
**LOY**	0.674									
**SAT**	0.659	0.549								
**CUS**	0.653	0.646	0.642							
**FUL**	0.604	0.585	0.758	0.772						
**PEN**	0.767	0.836	0.681	0.768	0.670					
**PER**	0.710	0.789	0.754	0.738	0.755	0.811				
**EFF**	0.762	0.82	0.693	0.700	0.686	0.835	0.875			
**SEC**	0.518	0.609	0.656	0.738	0.818	0.614	0.664	0.549		
**SYD**	0.614	0.634	0.495	0.726	0.663	0.687	0.589	0.655	0.596	

### 5.3 Measurement model: second-order construct with formative indicators

**Fig. 3:**
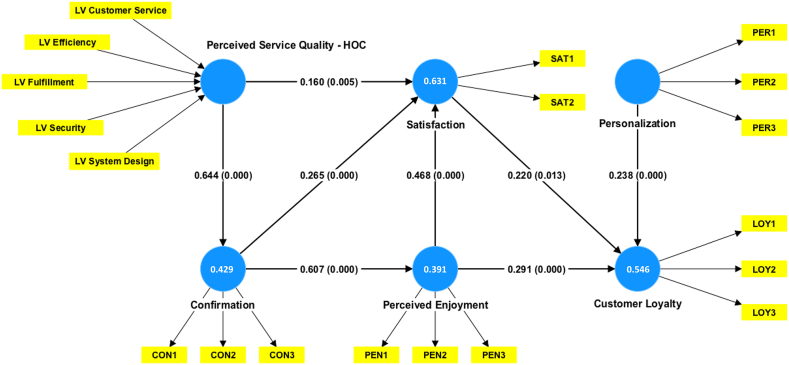
The HOCs structural model.

In the second stage, the latent variable scores of PSQ dimensions served as manifest variables for PSQ construct in the HOC measurement model (see Fig. 3). The multicollinearity test conducted on the PSQ dimensions revealed that all VIF values remained below the critical level of 5 and the conservative threshold of 3 [66,68], indicating that multicollinearity among PSQ dimensions was not an issue. The results of the significance test in Table 7 show that the security dimension (LV security) was not statistically significant, whereas the other four dimensions showed statistical significance (*p<0.05*). It is important to note that a lack of statistical significance for an indicator does not automatically imply poor measurement model quality [69]. To assess the absolute contribution of a formative indicator, researchers should consider its outer loading value. For LV security, the outer loading was 0.687 (Table 8), surpassing the threshold of 0.5 [69]. This result indicates security's absolute importance for the PSQ construct, thus justifying the retention of this dimension. Based on the results of the measurement model tests, the formative construct was deemed suitable for testing the structural model.

**Table 7:** Content validity of the formative measurement construct: VIFs, weight significances, and bootstrap tests.

	VIF	Weights	*t-Stat*	*p-Value*	Lower 2.5%	Upper 97.5%
LV System Design → PSQ	1.465	0.341	4.473	0.000	0.186	0.491
LV Fulfillment → PSQ	2.225	0.226	2.418	0.008	0.079	0.312
LV Security → PSQ	2.088	0.189	1.863	0.062	−0.015	0.382
LV Customer Service → PSQ	1.632	0.447	5.952	0.001	0.292	0.588
LV Efficiency → PSQ	1.795	0.271	3.204	0.001	0.098	0.429

**Table 8:** The outer loadings of PSQ dimensions.

	Original sample (O)	Sample mean (M)	*t-Stat*	*p-Value*
LV System Design → PSQ	0.687	0.681	13.089	0.000
LV Fulfillment → PSQ	0.768	0.760	21.046	0.000
LV Security → PSQ	0.773	0.765	16.784	0.000
LV Customer Service → PSQ	0.840	0.840	39.732	0.000
LV Efficiency → PSQ	0.829	0.822	16.470	0.000

### 5.4 Structural model and hypotheses test

The structural model assessment consisted of the coefficient of determination (*R*^*2*^), the effect sizes (*f*^*2*^), and the path coefficient estimates. The *R*^*2*^ values for constructs Confirmation, Customer Loyalty, Perceived Enjoyment, and Satisfaction were 0.429, 0.546, 0.391, and 0.631, respectively. Table 9 presents the effect sizes and path coefficient estimates. All hypotheses were supported as summarized in Fig. 3.

**Table 9:** Structural relationship test results.

Hypothesis	*f*^*2*^	Path coefficient^1^ (Sig. value)	*t-Stat*	Conclusion
H1: PSQ → satisfaction	0.053	0.160** (0.005)	2.813	Supported
H2: PSQ → confirmation	0.708	0.644*** (0.000)	16.812	Supported
H3: Confirmation → satisfaction	0.194	0.265*** (0.000)	4.299	Supported
H4: Confirmation → perceived enjoyment	0.584	0.607*** (0.000)	15.539	Supported
H5: Perceived enjoyment → satisfaction	0.304	0.468*** (0.000)	7.682	Supported
H6: Perceived enjoyment → customer loyalty	0.164	0.291*** (0.000)	4.075	Supported
H7: Satisfaction → customer loyalty	0.036	0.220* (0.013)	2.472	Supported
H8: Personalization → customer loyalty	0.157	0.238*** (0.000)	3.867	Supported

Note: ^1^ *p < 0.05, **p < 0.01, ***p < 0.001.

### 5.5 Hypotheses testing using classification algorithms

Previous studies have utilized machine learning classification algorithms to validate predictive models [[79], [80], [81], [82]]. This study used five classifiers (i.e., Naïve Bayes, SMO, IBk, OneR, and Random Forest) to test the relationships among the constructs in the ECLM model in 10-fold cross-validation using WEKA version 3.8 [83]. Based on the research model shown in Fig. 1, we developed four classifier models: (1) predicting confirmation by perceived service quality; (2) predicting perceived enjoyment by confirmation; (3) predicting customer satisfaction by perceived service quality, confirmation, and perceived enjoyment; and (4) predicting customer loyalty by customer satisfaction, perceived enjoyment, and personalization. Since the dataset in this study was imbalanced, best performance classifiers are analyzed based on the F1 score and ROC area. The F1 score combines precision and recall, providing a single measure that balances their trade-offs. Its value ranges between 0 and 1, with higher values indicating better performance. A high F1 score implies a good balance between precision and recall, with high prediction accuracy. The ROC area provides a summary metric that quantifies the extent to which the model distinguishes between positive and negative instances across different threshold settings. Its range is between 0 and 1, with higher values indicating a stronger discriminatory power. High values of the F1 score and ROC area of the best performance classifiers indicate support for the hypothesis testing results [74,79].

In predicting confirmation by perceived service quality (see Table 10), the F1 scores for SMO and Random Forest are the highest among the classifiers at 0.876 and 0.874, respectively. High value of F1 score implies a good balance between precision and recall, with high correctness in its predictions. Based on ROC area, all classifiers have values above 0.8, indicating a high ROC area, with Naïve Bayes as the highest performance. A high ROC Area suggests that the model has a high true positive rate and a low false positive rate. It signifies a high level of confidence in the model's ability to differentiate between different classes. Based on the best performance classifiers results, it provides support to H2 acceptance. The results in Table 11 show that Random Forest classifier had the highest F1 score and ROC area at 0.875 and 0.901, respectively. Hence, this result also supports the acceptance of H4.

**Table 10:** Predicting confirmation by perceived service quality.

Classifier	Accuracy^a^	Precision	Recall	F1 Score	ROC Area
Naïve Bayes	86.283	0.860	0.863	0.860	0.888
SMO^b^	87.906	0.877	0.879	0.876	0.827
iBK^c^	84.849	0.846	0.848	0.841	0.837
OneR	86.700	0.865	0.867	0.863	0.814
Random Forrest	87.758	0.875	0.878	0.874	0.877

Note.

a accuracy: correctly classified instances (%).

b SMO: Sequential Modeling Optimization.

c IBk: Instance based learning.

**Table 11:** Predicting perceived enjoyment by confirmation.

Classifier	Accuracy^a^	Precision	Recall	F1 Score	ROC Area
Naïve Bayes	86.431	0.886	0.864	0.873	0.820
SMO^b^	88.053	0.851	0.881	0.852	0.824
iBK^c^	86.326	0.831	0.867	0.841	0.755
OneR	87.574	0.840	0.876	0.848	0.562
Random Forrest	88.421	0.869	0.884	0.875	0.901

Note.

a accuracy: correctly classified instances (%).

b SMO: Sequential Modeling Optimization.

c IBk: Instance based learning.

When predicting customer satisfaction by perceived service quality, confirmation, and perceived enjoyment, SMO has the highest performance in accuracy (88.235%) and F1 Score (0.874), as reported in Table 12. Based on the ROC Area, Naïve Bayes outperforms other classifiers at 0.858. Therefore, these results confirm hypotheses H1, H3, and H5. Finally, the results in Table 13 show that SMO outperforms other classifiers in predicting customer loyalty by perceived enjoyment, customer satisfaction, and personalization at 90.266% correctly classified instances and 0.891 F1 score. However, Random Forest has the highest ROC area score at 0.888, Accordingly, these results support the acceptance of H6, H7, and H8.

**Table 12:** Predicting customer satisfaction by perceived service quality, confirmation, and perceived enjoyment.

Classifier	Accuracy^a^	Precision	Recall	F1 Score	ROC Area
Naïve Bayes	83.481	0.841	0.835	0.838	0.858
SMO^b^	88.235	0.875	0.882	0.874	0.751
iBK^c^	80.531	0.794	0.805	0.798	0.763
OneR	83.186	0.820	0.832	0.823	0.702
Random Forrest	82.743	0.820	0.827	0.823	0.839

Note.

a accuracy: correctly classified instances (%).

b SMO: Sequential Modeling Optimization.

c IBk: Instance based learning.

**Table 13:** Predicting customer loyalty by customer satisfaction, perceived enjoyment, and personalization.

Classifier	Accuracy^a^	Precision	Recall	F1 Score	ROC Area
Naïve Bayes	88.201	0.870	0.882	0.870	0.793
SMO^b^	90.266	0.892	0.903	0.891	0.869
iBK^c^	87.446	0.880	0.874	0.877	0.821
OneR	89.676	0.890	0.897	0.893	0.740
Random Forrest	88.235	0.895	0.882	0.887	0.888

Note.

a accuracy: correctly classified instances (%).

b SMO: Sequential Modeling Optimization.

c IBk: Instance based learning.

## 6 Discussion

Despite the significant growth of e-marketplace's customers and transactions, preserving customer loyalty remains a great challenge, because customers are becoming price-conscious. This study examines factors contributing to customer loyalty in the e-marketplace industry and understanding how customer loyalty works are crucial to business growth. Results and findings from this study show that the proposed E-marketplace Customer Loyalty Model can highlight customer behavior in using e-marketplace. The discussion is presented in three themes: (1) factors affecting customer loyalty to use e-marketplaces, (2) implications, and (3) limitations and future directions.

### 6.1 Factors affecting the customer loyalty to use E-marketplace

Through the conducted study, it was found that system design, fulfillment, customer service, and efficiency dimensions have relative contributions to PSQ, while the security dimension has an absolute contribution to PSQ. This suggests that the security dimension may make a relatively small contribution to the PSQ compared to the other dimensions. This finding can be attributed to the general behavior of Indonesian customers toward security risks. Although cybersecurity awareness has increased, their perception of security risks is generally low, and their level of confidence in providing personal information is high. Based on demographic data, approximately 80% of the respondents in this study were aged between 18 and 35 years. Young customers show highly impulsive buying behavior tendencies [84]. When impulsive urges drive a buying decision, customers show a higher intention to shop and tend to neglect security risks.

The supporting H1 and H2 verify the roles of PSQ as a key factor of customer satisfaction and confirmation, which is consistent with previous studies that reported that the customers' perception of system quality influenced the confirmation between their expectations and the actual use [18,35]. When an e-marketplace performance exceeds the customer's original expectation, the positive confirmation increases post-adoption satisfaction. Interestingly, albeit the positive correlation with customer satisfaction, the effect size of PSQ is considered minimal in this study. Since online shopping has become a habit for most customers, they consider excellent service quality as a standard, not a luxury. Their expectations extend from the fulfillment of utilitarian-oriented goals, such as purchasing items efficiently, to the inclusion of hedonic-oriented goals, such as enjoyment and entertainment, as shown in H5 acceptance. The fulfillment of hedonic goals enhances the customer's satisfaction and accelerates acceptance as it accommodates the fun and hassle-free aspects of using the system/technology. Based on the path coefficient estimates, the fulfillment of hedonic values through perceived enjoyment has a greater impact on customer satisfaction (path coefficient = 0.468, *p* < 0.001) than utilitarian values in PSQ (path coefficient = 0.160, *p* < 0.05).

The study also verifies the influence of the extent of confirmation on customer satisfaction and perceived enjoyment, as shown by H3 and H4 acceptance. This implies that customers experience a greater extent of utilitarian and hedonic values when their expectations toward e-marketplaces are established. The positive confirmation then induces higher levels of satisfaction and perceived enjoyment. Subsequently, the results also show that customer satisfaction and perceived enjoyment are significant predictors of customer loyalty, as shown in H6 and H7, respectively. When customers are satisfied with service quality, they consider further usage of the e-marketplace and respond to loyalty programs. Although a satisfied customer is not always a loyal customer, studies have shown that customer satisfaction is key to securing customer loyalty [13,18,26]. Similarly, the fulfillment of hedonic values through perceived enjoyment supports customer loyalty in the e-marketplace. Perceived enjoyment is a crucial determinant of the continuous use of a system/technology that serves both utilitarian and hedonic values [18,37,85]. Lastly, the supporting H8 confirms the effect of personalization on customer loyalty in the e-marketplace. This result strengthens the findings of [38,46]. Personalization simplifies the purchasing process as the system automates frequent tasks, such as filling out personal and financial details, and it drives more transactions from the recommendation of relevant items and offerings that match customers' needs. Personalization also generates positive emotions [86], such as the feeling of being special and recognized, which can further strengthen customers’ attachment to a particular e-marketplace.

### 6.2 Theoretical and practical implications

The results and findings of this study provide theoretical and practical implications for researchers, e-marketplace providers, and developers. For theoretical implications, this study contributes to the existing literature by developing a new integrated model, the E-marketplace Customer Loyalty Model, for specifically predicting customer loyalty in the e-marketplace. The ECLM incorporates ECT constructs, perceived service quality dimensions, efficiency dimension, The User Acceptance of Hedonic Information Systems construct (i.e., perceived enjoyment), and the personalization construct to examine factors contributing to customer loyalty. The relationships among the constructs in the hierarchical component model of ECLM were validated using PLS-SEM and machine learning classification. The structural model's outcomes served as the prediction model's foundation. The best performance classifier in each prediction model is able to accurately predict instances with accuracies between 87.906% and 90.266%. Second, this study also presents further evidence that the fulfillment of hedonic values generates greater impacts on customer satisfaction in using e-marketplaces. The e-marketplace has evolved from simply a selling and buying platform to a super app encompassing Omni-features; hence customers have extended their expectations from mostly utilitarian-oriented goals to hedonic-oriented ones. Third, this study confirms the relative contribution of the efficiency dimension to PSQ. This finding could provide further consideration for extending e-service quality dimensions in future research.

From an industrial perspective, the results and findings of this study can be used as a guideline to develop features and improvements that can enhance customer loyalty. One of the most challenging aspects of building customer loyalty is keeping engagement rates high. E-marketplace providers should go beyond the purchasing cycle and provide rewards for non-transactional activities. Examples include additional points or benefits for writing reviews and sharing items on social media to help positive word of mouth; incentives for frequent logins and exploration of recommended content. Another strategy to promote customer engagement is addressing hedonic-oriented goals in e-marketplaces. Novelties and interactive elements, such as gamification and intelligent personalization, evoke positive experiences and deliver hedonic values in e-marketplaces. Gamification can be implemented in any customer interaction, including non-transactional and post-purchase activities. Second, incorporating cultural and local values in characterizing the customer behavior is needed to formulate more suitable customer loyalty programs. For example, online word of mouth and referral programs are likely to generate stronger effects in highly collectivist societies [87], where team cohesiveness is encouraged, and individuals are expected to be part of teams or organizations. Next, strengthening fundamental and proven strategies, and making purchases simple and convenient can improve customer experience. The findings from this study show that despite the complexity of the current e-marketplaces, customers still demand convenient and efficient shopping, such as a one-click checkout feature, instant and fast delivery, relevant items recommendation, and a lite version of e-marketplace application. Finally, the e-marketplace should incorporate comprehensive customer research to deliver robust and sustainable loyalty programs.

### 6.3 Limitations and direction for future studies

This study exhibits several limitations that could be addressed in future research. First, the study outcomes may be difficult to generalize. The majority of participants in this study ages between 18 and 35 years at 80%, as shown in the demographics profile in Table 1. The age distribution is in line with the recent survey reporting that millennials and Z generations dominated e-commerce transactions as a buyer at 85% of the total transactions in Indonesian e-marketplaces [88]. This study was conducted in a local context, both participants and e-marketplace platforms are based in Indonesia, hence the results may differ in other countries and regions. Second, the ECLM may not consider important relationships among included constructs, for example, there may be significant effects of PSQ dimensions on customer loyalty. Similarly, there may be other notable information systems theories and models that are more suitable to explain customer behavior in using e-marketplaces, such as the users and gratification theory, mobile service quality, and User Acceptance of the Hedonic Information Systems model. Future studies should consider exploring other relationships and incorporating consumer behavior and information systems theories.

Since the usage of e-marketplace has included more hedonic-oriented goals, the research model should incorporate constructs to examine usage and continuance intention for hedonic information systems, such as hedonic motivation, customer experience, word of mouth, and achievement. Future studies should also investigate the potential moderating effects among constructs. Finally, there are distinctive online shopping behaviors across age groups, genders, and tiers of membership. Therefore, future researchers are recommended to include customer demographics as covariates in statistical analyses.

## 7 Conclusion

This study investigates factors contributing to customer loyalty as a buyer in the Indonesian e-marketplace. The proposed research model, the E-marketplace Customer Loyalty Model, integrates PSQ dimensions, expectation-confirmation model constructs, the User Acceptance of Hedonic Information Systems, and personalization. Employing two different approaches (Partial least squares Hierarchical Component Model and machine learning classification algorithms) in hypothesis testing enables the development of a robust model and provides further evidence to support the hypothesis results. Since online shopping has become a regular activity for most customers, the customer goals of using e-marketplaces have been extended to include more hedonic-oriented goals. The results suggest that the fulfillment of hedonic values through perceived enjoyment generates a greater impact on customer satisfaction. The result also revealed the positive influences of customer satisfaction and personalization on customer loyalty. Based on results and findings, this study offers practical implications as a guide for e-marketplace providers to design robust and sustainable customer loyalty programs.

## Author contribution statement

Ira Puspitasari: Conceived and designed the experiments; Performed the experiments; Analyzed and interpreted the data; Wrote the paper.

Febdian Rusydi: Conceived and designed the experiments; Analyzed and interpreted the data; Wrote the paper.

Nania Nuzulita: Performed the experiments; Contributed reagents, materials, analysis tools or data.

Chin-Sung Hsiao: Conceived and designed the experiments; Wrote the paper.

## Data availability statement

Data will be made available on request.

## Declaration of competing interest

The authors declare that they have no known competing financial interests or personal relationships that could have appeared to influence the work reported in this paper.
