# Supplementary material for: Investigating the role of utilitarian and hedonic goals in characterizing customer loyalty in E-marketplaces
Source: Heliyon. 2023 Aug 16;9(8):e19193. doi: 10.1016/j.heliyon.2023.e19193 (PMC10462820; doi:10.1016/j.heliyon.2023.e19193)
Supplement: Multimedia component 1 [file mmc1.pdf]

Supplementary Material

Survey (English Translation)

Dear participant,

Recent surveys and studies have reported an increase in e-marketplace use in Indonesia. This surge is attributed to a growing youth and middle-class population, and the widespread adoption of digital payment services. The COVID-19 pandemic has contributed to an increase in transactions on major e-marketplace platforms. Many users shifted to online platforms since most provinces and cities imposed large-scale social restrictions during the COVID-19 pandemic, and they continued using the e-marketplace after the pandemic slowed down.

You are invited to participate in a survey on the study about the customer behavior in e-marketplace. E-marketplaces offer a digital space for buyers and sellers to trade and conduct various types of business transactions. Some popular e-marketplace platforms in Indonesia are Tokopedia, Shopee, Bukalapak, Blibli, and Lazada. The goal of this study is to investigate the factors affecting customer behavior and loyalty to e-marketplaces. Customer refers to buyer who purchases products and services from official stores and/or sellers in e-marketplaces.

If you are 18 years old or older and have purchased items from at least one Indonesian-based e-marketplace on the web or mobile applications, please take a few minutes about your perception of the e-marketplace. Your participation in this online survey is completely voluntary. You may withdraw at any time prior to the completion of this survey by simply abandoning this survey. Your responses will be confidential and will be used only for research purposes.

Thank you very much for your participation.

Demographic Profile

Gender	: ☐ Male ☐ Female
Age	: ☐ 18 – 25 ☐ 26 - 35 ☐ 36 - 45 ☐ 46 - 55 ☐ > 55
Occupation	: ☐ Student: high school, undergraduate, graduate<br>☐ Civil servant<br>☐ Private employee<br>☐ Professional<br>☐ Entrepreneur<br>☐ Homemaker<br>☐ Other: ……………………………........
Domicile	: ☐ The Greater Jakarta, area including Jakarta, Bogor, Depok, Tangerang, and Bekasi cities<br>☐ Java (outside The Greater Jakarta) and Madura<br>☐ Bali and Nusa Tenggara<br>☐ Sumatra<br>☐ Kalimantan<br>☐ Sulawesi<br>☐ Maluku and Papua<br>☐ Other: ……………………………........

E-marketplace Profile Usage

Please select **all** e-marketplace platforms on which you have made at least one purchase.

☐ Tokopedia
☐ Shopee
☐ Bukalapak
☐ Lazada
☐ Blibli
☐ Other (please specify): ………………………………………………………………………………

Please select **one** e-marketplace platform that you have used the most.

☐ Tokopedia
☐ Shopee
☐ Bukalapak
☐ Lazada
☐ Blibli
☐ Other (please specify): ………………………………………………………………………………

E-marketplace period of use:

☐ less than 1 year
☐ 1 – 3 years
☐ 4 – 6 years
☐ more than 6 years

E-marketplace frequency of use:

☐ 1 – 5 times per month
☐ 6 – 10 times per month
☐ 11 – 15 times per month
☐ > 15 times per month
☐ As the need arises

Shopping activities in e-marketplaces (please select all activities that apply):

☐ Purchase items
☐ Top-up prepaid credits and pay bills
☐ Purchase tickets for travel and entertainment
☐ Access gamification features for additional benefits (e.g., discount, coupon, and cashback)
☐ Watch entertainment content for additional benefits (e.g., discount, coupon, and cashback)

Reason(s) for shopping in e-marketplaces (please select all answers that apply):

☐ Promotional campaigns: discount, cashback, and shipping fee reduction
☐ Ease of use
☐ Completeness of features
☐ Aesthetics design and clear navigation
☐ Brand ambassadors
☐ Other (please specify): ………………………………………………………………………………

Perception as a Customer on Participation in E-marketplaces

Please check (√) **one answer** for each statement that corresponds most closely to your agreement/disagreement based on the following scale:

1: strongly disagree; 2: disagree; 3: somewhat disagree; 4: somewhat agree; 5: agree; 6: strongly agree

Question	1	2	3	4	5	6
1. The use of the e-marketplace is clear and understandable.						
2. I can complete my purchase order easily, from searching a product, making a payment, to confirming transaction.						
3. It is easy to navigate the interface of e-marketplace website/application and find items / features that I am looking for.						
4. The e-marketplace ships orders within a reasonable timeframe.						
5. The logistics system of this e-marketplace provides enough options for me.						
6. The e-marketplace provides a guarantee for order fulfilment.						
7. The e-marketplace is truthful about its offers (e.g., price discount, cashback, and shipping fee reduction).						
8. The e-marketplace protects my financial information.						
9. The e-marketplace protects my personal information, such as personal details and purchasing behavior.						
10. The e-marketplace system has adequate security features.						
11. The e-marketplace provides a dedicated customer support to attend to any questions or comments that I might have.						
12. The e-marketplace resolves my problems promptly.						
13. The e-marketplace provides convenient options for returning items.						
14. The e-marketplace system loads its content quickly.						
15. The e-marketplace response to my actions quickly.						
16. The e-marketplace system is always available and ready to perform transactions whenever I access the system.						
17. The overall quality of the service provided by this e-marketplace is excellent.						
18. The overall quality of the e-marketplace system (website or application) is excellent.						

Question	1	2	3	4	5	6
19. My experience with using the e-marketplace is better than what I expected.						
20. The service level provided by the e-marketplace is better than what I expected.						
21. The majority of my expectations from using e-marketplace are confirmed.						
22. I enjoy exploring the e-marketplace features (e.g., exploring the flash sale, participating in a mission for rewards, using vouchers and coupons for additional benefits).						
23. I enjoy shopping in the e-marketplace.						
24. Overall, my experiences of using the e-marketplace are enjoyable.						
25. The e-marketplace always meets my needs.						
26. Overall, I am satisfied with this e-marketplace.						
27. The e-marketplace suggests items that suit my preferences.						
28. The promotion and advertisements in the e-marketplace are relevant to my shopping needs.						
29. The personalization provided by the e-marketplace benefits me.						
30. I recommend this e-marketplace to others (referrals).						
31. I am likely to continue to use the marketplace in the future (repeat purchase).						
32. I intend to continue using the e-marketplace rather than discontinue its use.						
